# Is the Dental Profession Crowded?

**Published:** 1878-05

**Authors:** 


### ARTICLE IV.

### *Is the Dental Profession Crowded?*

BY PROF. HODGKIN.

The young man who contemplates entering the ranks of the profession of Dentistry may very seriously ask this question. Of course the ready answer is the old one, "Not in the upper stories." But is it now, really? And really to look at the multitude of signs bearing the title of "Dentist," and ranging all the way from the modest and unobtrusive door plate to the blazoned beacon which in letters of big gilt heralds the shop of the "gas man," which are seen in our cities and adorn our avenues, one might well ask, is the profession crowded?

Comparison may help us. Prof. Pepper, of the Pennsylvania University, Medical Department, in an address at the opening of the last session of that school, gives some interesting statistics to show the abundance of Doctors of Medicine. Here, in the United States, we have one doctor to every 600 souls; in France, one to every 1814 souls; in Great Britain, one to every 1672 souls: in Germany, one to every 3000 souls; in Austria, one to every 2500 souls. That is to say in Europe there is about one medical man to every 2250 of population, or about one fourth the number that "prevail" in this free country, where anybody who wants to be a doctor can be one if he can purchase the tickets. Moreover in those embarrassing countries of Europe only about 900 a year of new doctors are made, while in this new world we turn out 3000 annually.

We are strongly tempted to go on and write something harsh about our medical relatives, for certainly the opportunity offers; but we are hardly safe to throw stones, living in these houses of glass of ours, and besides the theme will keep. It is hard to get at the number of dentists in the United States. The total is roughly estimated at about 10,000, which would be about one to every 4500 of the population. This is a large number for any dentist to have the care of and would be with difficulty attended to, but we must remember two facts: first, that this number embraces the infantile population, and half of these perish long before they are the subjects of the dentist's science, passing away before the age of six years, and only requiring his help for the occasional extraction of an aching deciduous tooth.

How many of the 2200 and odd, that get their permanent teeth, are what might be called patients? We may safely say that one half of these are only subjects for extraction, not filling, and their subsequent assignment to the dental mechanician, excludes them from classification with those we may fairly call patients. So that after all we will have hardly a thousand apiece left. Of this one thousand, how many are really patients in the sense in which we would delight to use the word? Not many. A few visit the office regularly to see if anything is wrong; a large number postpone the day of their coming until driven by pain; a blessed, very few don't need us at all,—and really we have not got many left to operate for. Of these, the men who by talent, or cheek, or fortuitous circumstance get in the front, obtain control of the large number, and what is left is scarcely sufficient to keep the rest in life's needs.

But the profession is only *seemingly* crowded. We who teach in the schools, know this; we know that the classes who sit winter by winter to hear the lectures, contain many men who will not be at all in the way of any earnest man.

For our part we love to teach, or try to teach these young men. They are very attentive, respectful auditors. That they do not learn dentistry is the fault of—whom? Given the facilities, lectures, demonstrations, access to the anatomical and pathological curiosity shops, free scope among the poor wretches who flock to the Infirmaries, —with all these, one in every ten or twenty is a success in after life.

They fail to do well for themselves or their patients, they damage their profession in the eyes of its kindred callings, they are cripples in a race with the few strong and swift.

Primarily, the fault is with those who take students, not with the colleges. The dentist who finds his mechanical work crowding him, takes a young man, or boy perhaps, and puts him in his laboratory. He, (the dentist) seldom has more in his mind than that he wants help and must get it cheaply. He pays the boy nothing, but usually gets a few hundreds of dollars from him. The student contracts to stay for no special length of time, and probably sets up for himself in a year or two. But the worst feature is, that as a rule no standard of qualifications is exacted, whether the student can read or write, whether he be boor or gentleman, matters not, so he can grind teeth and polish plates.

But the whole subject is too familiar for repetition. If dentists would take only such students as are qualified by nature and training, such students as possess a good educacation, such as is obtained in our grammar schools at least; and if they would give them a chance to *study*,—not the temperature at which rubber is most readily hardened, but study their profession at the chair side, and if they would see that this was done for at least three years, and then see that they attended college lectures two years, a different state of affairs would be had, and perhaps the profession would begin to be crowded with men who could fairly compete with the ablest. But to any young man who will first fill his mind with knowledge, as he can best obtain it in some literary institution, and then study dentistry for at least five years, following that course with a graduation at a *good* medical school, and then start out, he will not be likely to find the position he takes in the profession he has chosen, at all crowded.

We have written this in vain if the reader does not gather from it that the felt wants of dentistry are preliminary training of a general sort, and abundant special training of a special sort. In a word, that the trouble is not that there are too many dentists, so much as that the many have no adequate training to fit them for their chosen profession. The remedy for this lies mainly with the profession, not with the colleges. These are constantly having forced on them students whose capacities might perhaps be developed by years, but which cannot certainly be brought out in the few months of training in the dental schools. If preceptors will take no students except such as are possessed of a fair share of mental training, and will keep them by them until they know something, and then see to it that they attend two full courses at a dental college, we shall see a different state of affairs very soon.

---

